# Objective Evaluation of Risk Factors for Radiation Dermatitis in Whole-Breast Irradiation Using the Spectrophotometric L*a*b Color-Space

**DOI:** 10.3390/cancers12092444

**Published:** 2020-08-28

**Authors:** Alexander M. C. Böhner, David Koch, Frederic Carsten Schmeel, Fred Röhner, Felix Schoroth, Gustavo R. Sarria, Alina-Valik Abramian, Brigitta Gertrud Baumert, Frank Anton Giordano, Leonard Christopher Schmeel

**Affiliations:** 1 Department of Radiation Oncology, University Hospital Bonn, University of Bonn, Venusberg Campus 1, 53127 Bonn, Germany; alexander.boehner@ukbonn.de (A.M.C.B.); david.koch@ukbonn.de (D.K.); fred.roehner@ukbonn.de (F.R.); felix.schoroth@ukbonn.de (F.S.); gustavo.sarria@ukbonn.de (G.R.S.); frank.giordano@ukbonn.de (F.A.G.); 2 Institute for Experimental Immunology, University Hospital Bonn, University of Bonn, Venusberg Campus 1, 53127 Bonn, Germany; 3 Department of Neuroradiology, University Hospital Bonn, University of Bonn, Venusberg Campus 1, 53127 Bonn, Germany; carsten.schmeel@ukbonn.de; 4 Department of Gynecology and Obstetrics, Division of Senology, University Hospital Bonn, University of Bonn, Venusberg Campus 1, 53127 Bonn, Germany; alina.abramian@ukbonn.de; 5 Institute of Radiation Oncology, Graubuenden Cantonal Hospital, Loestr. 170, 7000 Chur, Switzerland; brigittagertrud.baumert@ksgr.ch

**Keywords:** radiation dermatitis, radiodermatitis, breast cancer, whole-breast irradiation, risk factor, spectrophotometry, radiotherapy, RISRAS, L*a*b* color-space

## Abstract

**Simple Summary:**

In this prospective study, radiation dermatitis severity of 142 Caucasian early breast cancer patients undergoing whole-breast irradiation was evaluated by physicians, the patients themselves and objective technical measurements. The primary aim and a substantial novelty of this study was to identify patient- and treatment-related risk factors for radiation dermatitis by using objective spectrophotometry: 24 patient or radiotherapy related parameters were evaluated as potential risk factors. Objective and significant risk factors for radiation dermatitis were the breast volume and the applied irradiation technique; a boost radiotherapy administration also showed a trend towards a slightly more severe radiation dermatitis. These results can help to identify those patients at increased risk of developing a severe radiation dermatitis, as susceptible patients may require special monitoring and timely treatment.

**Abstract:**

*Background*: Radiation-induced dermatitis (RID) is frequent in breast cancer patients undergoing radiotherapy (RT). Spectrophotometry (SP) is an objective and reliable tool for assessing RID severity. Despite intensive research efforts during the past decades, no sustainable prophylactic and treatment strategies have been found. Estimation of new and reevaluation of established risk factors leading to severe RID is therefore of major importance. *Methods*: 142 early breast cancer patients underwent whole-breast irradiation following breast-conserving surgery. RID was evaluated by physician-assessed Common Terminology Criteria of Adverse Events (CTCAE v4.03). Spectrophotometers provided additional semi quantification of RID using the L*a*b color-space. A total of 24 patient- and treatment-related parameters as well as subjective patient-assessed symptoms were analyzed. *Results*: Values for a*max strongly correlated with the assessment of RID severity by physicians. Breast volume, initial darker skin, boost administration, and treatment technique were identified as risk factors for severe RID. RID severity positively correlated with the patients’ perception of pain, burning, and reduction of everyday activities. *Conclusion*s: Physician-assessed RID gradings correlate with objective SP skin measurements. Treatment technique and high breast volumes were identified as objective and significant predictors of RID. Our data provide a solid benchmark for future studies on RID with objective SP.

## 1. Introduction

Radiation-induced dermatitis (RID) is one of the most common side effects during and following radiation therapy (RT) [1]. RID can impair the patients’ quality of life for several weeks, and may also necessitate an RT interruption to recover skin integrity [2]. Any interruption of the RT could, however, reduce the allover treatment efficacy and might thus result in higher tumor recurrence and impaired tumor control rates, as dose declines of 0.6 Gy were reported for each unexpected day of interruption [3,4]. The identification and evaluation of specific risk factors for RID is imperative to anticipate RID severity and to adjust the therapy accordingly. These adjustments are based on both the avoidance and mitigation strategies for RID alike [2,5,6].

In early breast cancer, breast-conserving therapy is standard of care as it facilitates improved cosmetic results [7]. To prevent local tumor recurrence, surgical excision is mandatorily combined with adjuvant whole-breast irradiation (WBI). Recent studies have additionally shown that this multimodal approach provides at least equal or even better tumor control than mastectomy [7,8]. WBI is therefore ideally suited for the investigation of RID, because it is a frequent treatment and RID affects almost all WBI patients, regardless of the irradiation technique or fractionation used.

Despite the frequency of radiation dermatitis in WBI patients, there is still no comprehensive and, importantly, objective severity classification. Two of the most widely used severity gradings are the Common Terminology Criteria of Adverse Events (CTCAE) and RTOG/EORTC systems which are based on the visible skin color and integrity defined by erythema and desquamation [9]. These gradings are both easy to use and mostly sufficient for daily clinical routines. However, considerable and significant intra- and inter-observer variabilities of such subjective RID gradings have been reported and it is therefore quite challenging to compare or rather interpret data obtained throughout different studies [9,10]. Consequently, objective and semiquantitative measurements are needed, particularly in the context of clinical RID research, in which even minimal differences are critical [9,11].

Such a technical approach to assess RID is spectrophotometry (SP) with which the skin color can be evaluated objectively and reliably by three distinct numerical parameters, the L*, a*, and b* value: together, these parameters define the so-called L*a*b color space in which every color in every lightness can be precisely depicted. In clinical routines and also in clinical trials it is, of course, desirable that the technical effort as well as the time spent on such measurements is reasonable [10]. Spectrophotometry takes these factors into account, as the application is simple and each measurement takes only seconds, which allows the assessment of major parts of the irradiated skin in approximately three minutes.

In this prospective study, RID severity of 142 early breast cancer patients undergoing WBI was evaluated by CTCAE gradings and SP. The primary aim and a substantial novelty of this study were to identify patient- and treatment-related risk factors for RID by using objective SP skin measurements. We also investigated the correlation of the subjective CTCAE score with the objective SP measurements; these subjective and objective data were additionally correlated with patient-reported symptoms using the Radiation-Induced Skin Reaction Assessment Scale (RISRAS) providing new insights into the relationship between subjectively experienced RID symptoms and objective, semi-quantitative skin measurements.

## 2. Results

### 2.1. Participants

142 women were prospectively enrolled from October 2017 to July 2019 and participated in this study. Patient data were gathered in our comprehensive academic cancer center, participating clinics, and radiation oncology community practices. The study design was previously approved by the local ethics committee (186/2016). Written informed consent was obtained by all participants. All analyzed patients completed WBI as intended. Patient characteristics are summarized in Table 1.

Inclusion criteria: WBI dose-fractionation regimen of 50 Gy in 25 fx (CF) or 40.05 Gy in 15 fx (HF), breast-preserving surgery, >18 years, nonsmoker. Exclusion criteria: neoadjuvant/adjuvant/concomitant chemotherapy, previous irradiation of the ipsilateral breast, nodal irradiation indicated, mastectomy, breast implants, any prior surgical procedures in the irradiation area, use of corticosteroids, active dermatitis or chronic dermatological disorders, tattoos within the treated integument, and patient refusal.

### 2.2. Visual Examinations and Spectrophotometry Correlate Positively with Maximum a* Values and Inversely for Maximum L* Values

**Figure 1:**
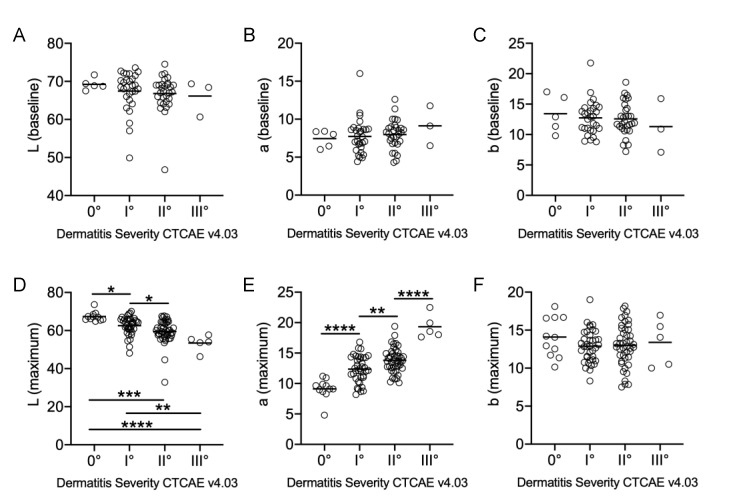
Spectrophotometric assessments of breasts at baseline (**A**–**C**) and after radiotherapy (RT) completion (**D**–**F**). All data points are categorized by their final dermatitis severity in accordance with Common Terminology Criteria of Adverse Events (CTCAE). *p* values were generated using one-way-ANOVA with * for *p* < 0.05, ** for *p* < 0.01, *** for *p* < 0.001 and **** for *p* < 0.0001. (**A**) Summary *p*-value determined by ANOVA was 0.73 (not significant). (**B**) Summary *p*-value determined by ANOVA was 0.68 (not significant). (**C**) Summary *p*-value determined by ANOVA was 0.77 (not significant). (**F**) Summary *p*-value determined by ANOVA was 0.55 (not significant). Median of individual groups depicted as a fine horizontal line.

Prior to the first RT, none of the women exhibited symptoms of dermatitis. The lightness of the skin was not linearly associated with severe RID (Figure 1A). Neither the skin color determined by a* or b* values were predictive of the final RID severity (Figure 1B,C). Throughout the course of RT, 90.1% of patients developed a RID (grade I–III). At treatment completion, physician-assessed RID severity and maximum a* values determined by SP and indicative of skin redness, correlated positively, and highly significantly (R^2^ = 0.4437; *p* < 0.0001). SP revealed a proportional decrease in skin lightness (L* values) (Figure 1D) and an increase in erythema (a* parameter) (Figure 1E). However, the b* parameter (blue-yellow axis) remained stable, regardless of RID severity (Figure 1F). A detailed statistical analysis of maximum L* and a* values sorted by RID severity gradings are provided in Table 2.

### 2.3. Patient-Related Risk Factors: Breast Volume and Darker Skin Are Risk Factors for Severe RID

**Figure 2:**
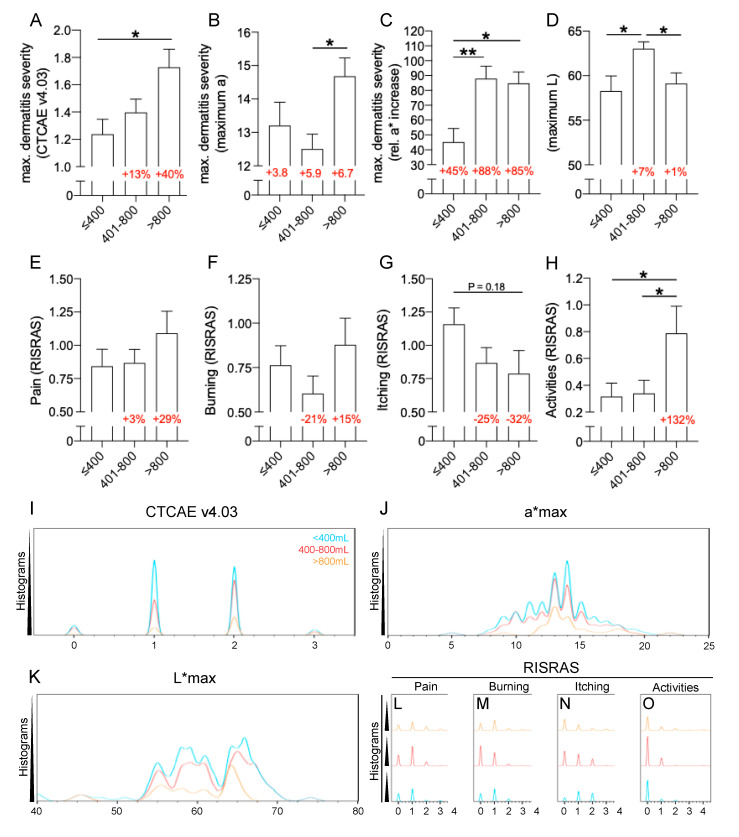
Breast volume is a positive risk factor for severe RID. (**A**) Maximum dermatitis severity was assessed by physicians according to CTCAE v4.03 in relation to different breast volumes. Percentages in red indicate the relative increase to the dermatitis severity for patients with a breast volume of <400 mL. (**B**) Maximum dermatitis severity is measured by spectrophotometry as maximum a* values in relation to different breast volumes. Numbers in red indicate the absolute increase to baseline a* values. (**C**) Maximum dermatitis severity is measured by spectrophotometry as relative a* value-increase (maximum to baseline) in relation to different breast volumes. Numbers in red indicate the relative increase to baseline a* values. (**D**) The lightness of the skin during RID enumerated by absolute L* maximum values obtained by SP. Red numbers indicate the alteration relative to patients with small breasts. (**E**–**H**) Subjective RISRAS assessment of RID related symptoms. (**E**) Pain (**F**) Burning (**G**) Itching (**H**) Reduction in everyday life activities. Red numbers indicate the alteration relative to patients with small breasts. (**I**–**O**) Distribution pattern analyses according to breast size <400 mL (light blue), 400–800 mL (red) and >800 mL (orange). (**I**) Visual CTCAE v4.03 grading (**J**) Maximum a* value (**K**) Maximum L* values (**L**) Pain assessed by RISRAS. (**M**) Burning assessed by RISRAS (**N**) Itching assessed by RISRAS (**O**) Reduction in everyday life activities. One-way-ANOVA has been conducted for significance detection with * for *p* < 0.05, ** for *p* < 0.01.

Breast volumes were predictive of severe RID, in particular when exceeding 800 mL (Figure 2). Initially, RID severity was linearly correlated with the absolute breast volume. It was calculated for both direct RID severity parameters, either by visual examination according to CTCAE v4.03 or via a* maximum values obtained by spectrophotometry. For CTCAE scores, the resulting regression yielded a statistically significant slope-deviance with *p* = 0.005 and R^2^ = 0.06. For spectrophotometrically obtained a*maximum values, the linear regression yielded a *p* = 0.051 and R^2^ = 0.05. Taking the strong positive correlation between CTCAE gradings and a*maximum values into account, the comparably low R^2^ values for the linear regressions with breast volumes were indicative of a skewed distribution pattern: consequently, subgroups were established allowing to draw more clinically relevant conclusions (Figure 2A–H).

Detailed distribution analyses further illustrate the skew distribution of changes of the breast skin or symptoms associated with RID (Figure 2I–O). The three groups divided the patients according to their breast volume into <400 mL (small), 400–800 mL (intermediate), and >800 mL (large). With this a priori categorization, large breasts yielded by average 40% more severe RID in CTCAE gradings in relation to small breasts (Figure 2A,I). The cohort with large breasts also yielded the highest a* maximum values (Figure 2B,J). The relative increase in a* values to baseline measurements were significantly elevated for intermediate and large breasts in contrast to small breasts, but no difference could be observed between intermediate and large breasts (Figure 2C). The lightness of the skin was, at the peak of RID, highest in intermediate breasts, while no difference between small and large breasts was apparent (Figure 2D,K). The depiction of individual symptoms contained in the RISRAS questionnaire has been calculated for breast volume subgroups (Figure 2E–H,L–O). While not significant, patients with large breasts reported >25% more pain than small or intermediate breasts (Figure 2E,L). For the burning sensation associated with RID, no specific impact of the breast volume could be observed (Figure 2F,M). Women with small breasts reported more intense itching following RT relative to larger breasts (Figure 2G,N), whilst patients with large breasts suffered from a RID related reduction in activities of daily living more than twice as severe as patients with small or intermediate breasts (Figure 2H,O).

**Figure 3:**
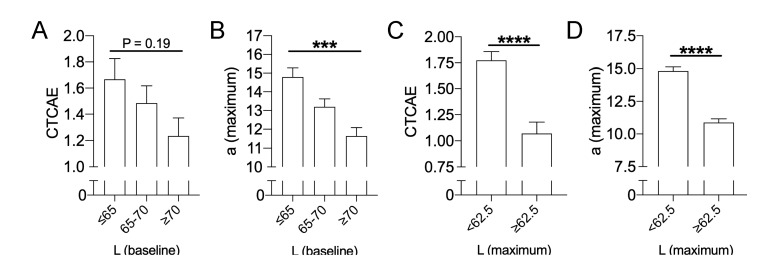
Initial dark skin is a positive risk factor for severe RID. (**A**) Maximum dermatitis severity assessed by physicians according to CTCAE v4.03 in relation to initial (pre-treatment) lightness of the skin. (**B**) Maximum dermatitis severity measured by spectrophotometry as maximum a* values in relation to initial (pre-treatment) lightness of the skin. (**C**) Maximum dermatitis severity assessed by physicians according to CTCAE v4.03 for either dark (L* max <62.5) or light (L* max ≥ 62.5) skin during RID. (**D**) Maximum dermatitis severity measured by spectrophotometry as maximum a* values for either dark (L* max < 62.5) or light (L* max ≥ 62.5) skin during RID. One-way-ANOVA has been conducted for significance detection in panels (**A** + **B**), unpaired *t*-tests for panels (**C** + **D**) with *** for *p* < 0.001, and **** for *p* < 0.0001.

After retrospective analysis conducted by sorting values according to CTCAE gradings (Figure 1), a deeper analysis was conducted aiming to extrapolate groups of patients at risk for severe RID. Following subgrouping, initial darker skin was predictive for severe RID, both measured by physicians according to CTCAE (Figure 3A) and reflectance spectrophotometry (Figure 3B): patients with an initial skin lightness below L* (baseline) ≤ 65 yielded a significantly higher a* maximum value during RID compared to patients presenting with lighter skin of L* (baseline) ≥ 70. After RT completion, the skin lightness (L* maximum) was also indicative for RID severity: for CTCAE scores (Figure 3C) as well as maximum a* values obtained by SP (Figure 3D), severe RID was associated with skin darkening.

The following patient-related factors did not correlate linearly with the severity of physician-assessed RID: patient age with *p* = 0.13 and R^2^ = 0.016; BMI with *p* = 0.23 and R^2^ = 0.010; sequential boost with *p* = 0.06 and R^2^ = 0.024; boost volume with *p* = 0.61 and R^2^ = 0.005; mean dose to PTV with *p* = 0.44 and R^2^ = 0.005; volume receiving > 107% of prescribed dose with *p* = 0.84 and R^2^ = 0.0004; HI-RTOG (maximum isodose in the target/reference isodose) with *p* = 0.82 and R^2^ = 0.0004; HI(D2-D98)/D50 with *p* = 0.71 and R^2^ = 0.001; L* (baseline) with *p* = 0.28 and R^2^ = 0.018; a* (baseline) with *p* = 0.29 and R^2^ = 0.017; b* (baseline) with *p* = 0.37 and R^2^ = 0.012; b* (maximum) with *p* = 0.52 and R^2^ = 0.004; rel. D_2%_ with *p* = 0.5635 and R^2^ = 0.002; rel. D_50%_ with *p* = 0.5532 and R^2^ = 0.002; rel. D_98%_ with *p* = 0.5595 and R^2^ = 0.002. Detailed results on all investigated parameters in relation to the CTCAE scoring, maximum L* value and maximum a* values are provided in Table 3.

### 2.4. Impact of a Sequential Boost and Treatment Technique on Maximum RID Severity

**Figure 4:**
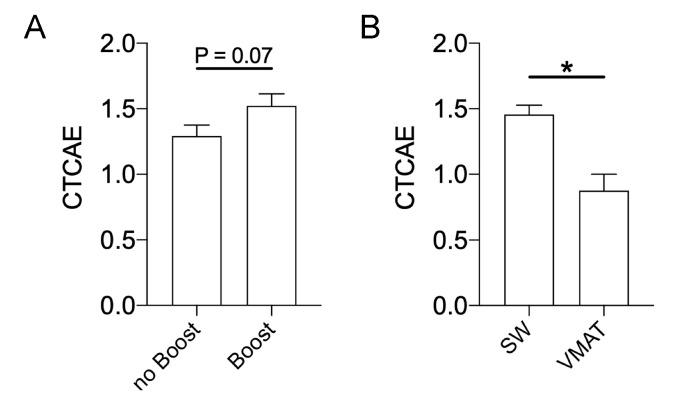
Impact of a sequential boost RT and treatment technique on RID severity. (**A**) Comparison between boost and no boost for RID severity. (**B**) Dermatitis severity is evaluated by different radiation techniques. Sliding window IMRT (SW) and volumetric-modulated arc therapy (VMAT) were compared by unpaired *t*-tests with * for *p* < 0.05.

A sequential boost RT led to a tendentially more severe RID (Figure 4A) with an absolute difference of 0.23 according to CTCAE scores. VMAT WBI resulted in significantly lower dermatitis grades compared with SW WBI with an absolute reduction of 0.60 in CTCAE score (Figure 4B).

### 2.5. Correlations between Physician Assessed RID Severity, Objective Skin Color, and Subjective Symptoms

**Figure 5:**
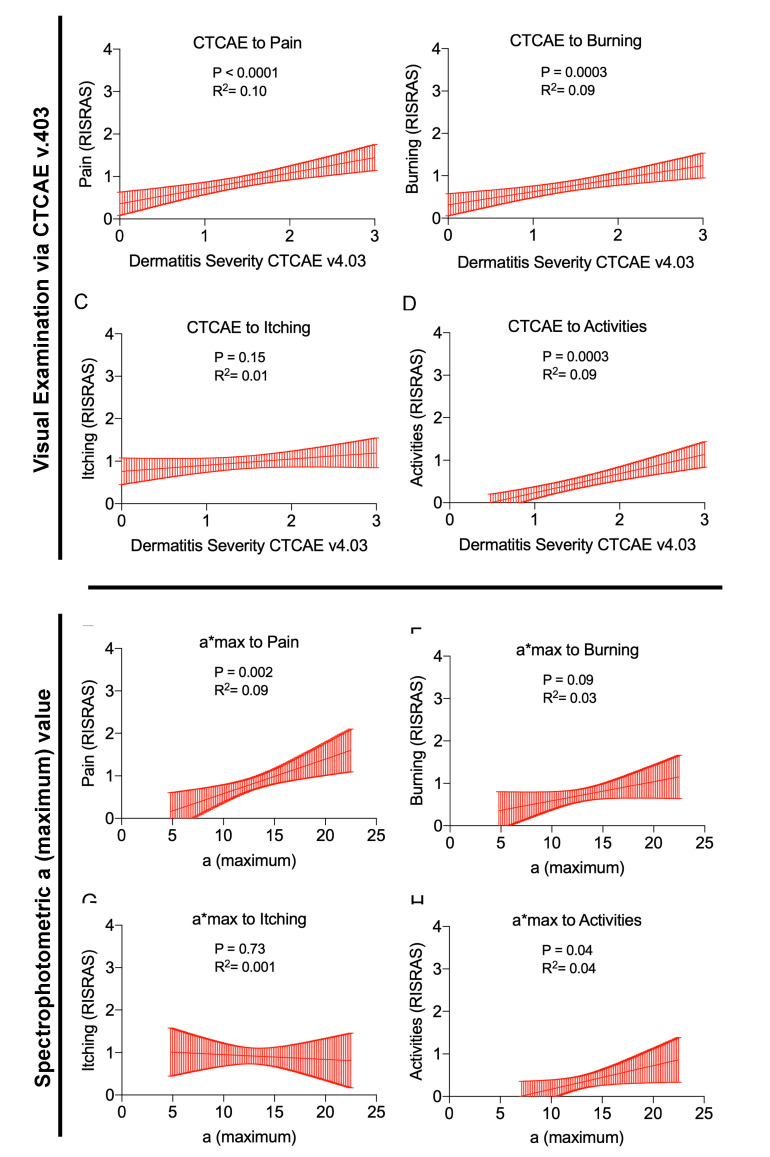
Correlations between RID severity and subjective symptoms. Straight red lines indicate the simple linear regressions, red shaded areas represent the 95% confidentiality intervals. The goodness of fit R^2^ and statistical slope deviance from zero (*p* values) are provided within each graph. (**A**–**D**) depict individual symptoms (RISRAS) correlated with physician-assessed CTCAE gradings. (**E**–**H**) Correlation of maximum a* values with patient-assessed RISRAS scores. (**A** + **E**) Pain of the irradiated skin area. (**B** + **F**) Burning sensation of the radiated skin area. (**C** + **G**) Itching sensation of the radiated skin area. (**D** + **H**) Reduction of everyday life activities due to RID.

To quantify the impact of RID severity on distinct subjective symptoms, patients enrolled in this study filled the RISRAS questionnaire. We performed statistical analyses based on CTCAE as well as maximum values for the green-red-axis within the L*a*b color-space (maximum a* value). With both assessment approaches, we found that an increasing RID severity proportionally increases the perception of pain and burning sensation (Figure 5A,B,E,F). However, itching was independent of the RID severity (Figure 5C,G). Activities of daily living were reduced in cases with severe RID only (Figure 5D,H). The goodness of fit for the linear regressions was better for the CTCAE score than for maximum a* values, while the latter was more discrete. Darkening of the skin due to RID, characterized by a decrease in maximum L* values, provided a reliable surrogate parameter for RID severity. Deviance from zero for the correlation of maximum L* to physician-assessed RID severity was *p* < 0.0001 and inversely correlated with symptoms measured by RISRAS (Radiation-Induced Skin Reaction Assessment Scale): pain (*p* = 0.0115); burning (*p* = 0.025) and reduction in activities of daily living (*p* = 0.0011). Maximum L* values did not correlate with the subjective perception of “Itching” (*p* = 0.8369). This finding is similar to RID assessments via maximum a* values and CTCAE. Similar to the maximum a* values and CTCAE v4.03 ratings, itching did not correlate with the maximum L* values (*p* = 0.8369).

## 3. Discussion

An important and frequent adverse reaction of adjuvant WBI is RID, often considerably impairing the patients’ quality of life during the course of WBI and up to weeks thereafter [1,2]. Generations of radiobiologists have devoted their efforts to investigate the underlying mechanisms of damage, the dose-effect relationship, and the different latencies of skin damage caused by ionizing radiation [12,13,14,15]. These valuable and comprehensive works constitute, inter alia, the basis of our current understanding of radiation-induced skin reactions. The development of therapeutic options was less successful: despite intensive research efforts, no sustainable RID treatment or prevention methods have been found yet. Strategies to assess RID and the identification of risk factors are therefore of major importance in RID research and patient care.

For decades, radiation oncologists used to rely on visually assessed and thus subjective criteria such as the CTCAE for the assessment of RID. Owing to the simple and quick application, these subjective gradings are widely accepted. However, such visual assessments were shown to be prone to considerable biases due to substantial intra- and inter-evaluator differences [6,9,11,16,17]. Especially when it comes to evaluating new therapeutic strategies for RID or the combination of radiotherapy with novel immunomodulatory agents, even minimal dermatitis differences are of interest, which in turn renders objective and precise assessments indispensable [11].

While initially developed for the dye and textile industry, SP was consequently utilized in the objective and reliable assessment of skin color differences. Feasibility studies showed that SP can determine even slight skin luminance and color differences which may not be perceived by a human observer or photographs [10,16,17,18]. Turesson et al. were among the first to use spectrophotometers for an accurate and objective radiation dermatitis evaluation already in the 1970s [19,20,21,22].

Along with objectivity, a continuous scale enumerating even slight changes in the skin color also allows us to draw precise conclusions on both the development of RID and the efficacy of specific treatments [2,5,11,22,23].

So far, our series yields the largest WBI patient cohort investigated by SP for dermatitis evaluation. As expected [10], physician-assessed CTCAE gradings positively correlated with objective SP in terms of maximum a* values, the indicator of erythema. The decrease in skin lightness quantified by the L* values was also strongly correlated with an increase in objective redness (maximum a* value) and the severity of CTCAE-based RID grading. Our analyses provide a solid benchmark for objective SP-based RID grading in Caucasian breast cancer patients: these data can be considered as reference values for comparison purposes in future RID research and with other ethnicities in this relevant field of clinical care.

Such an objective assessment of RID also serves an exact estimation of possible RID risk factors, as susceptible patients may require special monitoring and timely treatment [24]. Back between 1972 and 1985, Turesson et al. already pursued a comparable approach with spectrophotometry to identify risk factors for RID associated with electron irradiation of the internal mammary nodes [20]. We have recently shown by SP that RID frequency and severity are significantly reduced in hypofractionated vs. conventionally fractionated WBI [2]. Several previous, non-objective studies evaluated further possible risk factors for the development of RID, particularly in WBI [8,24,25,26]. Unfortunately, the respective results are inconsistent and often even contradictory–it may, therefore, appear rather difficult to derive any definitive risk factors for RID from the existing data. A recent study sums up this quandary [6]: RID was graded in 301 breasts following WBI by four radiation oncologists; statistical analysis hinted towards a younger age, boost irradiation, concurrent hormonal therapy and chemotherapy as possible risk factors for RID. However, no single risk factor was significant for all evaluators because of wide intra-evaluator variations and large inter-evaluator differences. In light of the aforementioned and varying study results, this quintessence was not entirely unexpected. Many authors, therefore, concluded that future studies should endeavor to meet the requirement for a more objective approach in the RID evaluation process [5,6,16,17].

Our series complies with this recommendation and is, to the best of our knowledge, the first to evaluate potential risk factors associated with RID in WBI based on objective SP. We found the breast volume to be a positive predictor of a more severe RID during WBI and confirmed this correlation by objective SP. Subgroup analyses revealed a significantly increased risk of a more severe RID in breasts exceeding 800 mL (visual grading), whilst SP already indicated significant erythema differences in breasts with more than 400 mL. A sequential tumor bed boost also induced a more severe RID, as measured by CTCAE and SP alike, while the aspired significance level was slightly exceeded. Large breast volumes and a boost administration have already been suggested as such predictors [8,25,26], but objective evidence to support this hypothesis was not yet available. Both factors should be considered carefully in WBI. 

Unexpectedly, a significant difference in RID severity was observed for different WBI treatment approaches: patients benefited from VMAT as compared with SW-IMRT. Conventional tangents or forward-planned 3D-conformal RT techniques were explicitly not used in our study since it was shown that modern techniques offer better dose conformity and homogeneity, and deliver lower doses to the ipsilateral lung and breast [24]. Up to now, only comparative planning studies between SW-IMRT and VMAT exist that still disagree on which treatment is better suited in terms of skin dose distribution [27,28,29,30]. This is not surprising, as the optimal treatment technique depends on the individual patient anatomy and RID is, beyond question, usually subordinate in relation to other organs at risk when choosing a WBI treatment plan. Results of such planning studies are also not necessarily transferable to the clinical effect in patients. Our observations and skin readings might, therefore, be important as they are statistically significant, even though the number of patients treated with VMAT was much lower than those treated with SW: our data could thus tempt to speculate that even minimally lower peak skin doses, as achieved by VMAT compared to SW-IMRT, result in reduced RID severity.

Another finding was that the pre-treatment skin color was not predictive of final RID severity in this Caucasian cohort. However, following stratification for pre-treatment skin lightness, objective SP indicated that those patients with darker skin developed significantly more intense erythema. Our objective measurements stand in contrast to previous, non-objective studies describing a relationship between light skin and RID susceptibility [19,26].

Additionally, we evaluated the impact of objective and subjective dermatitis severity on patient-experienced symptoms caused by RID. We determined whether higher objective skin color differences or physician-assessed severity gradings would affect the patients’ symptoms or vice versa. This may sound trivial at first glance and was thus not investigated yet, but it is highly relevant since substantial differences between patients’ and clinicians’ toxicity evaluations have been reported [7,31]. Pain, burning and a reduction in everyday life activities correlated positively to physician-recorded RID severity and objective SP. This did not apply to itching, which occurred in both mild and severe RID. These findings suggest that subjectively and objectively assessed skin alterations also correlate with patient symptoms but they cannot replace the patient interview.

Ideally, future objective studies in the field of RID research should also reflect patients’ genetic, epigenetic, and molecular profiles, since several promising relationships of tissue sensitivity towards radiotherapy have been described. Even though this kind of research is still in its infancy, we expect it to provide further clinically relevant insights into risk factors for radiotherapy-induced side effects [32,33,34].

## 4. Material and Methods

### 4.1. Radiation Technique

After 3D planning, patients were randomly assigned to either 50 Gy in 25 fractions (fx) or 40.05 Gy in 15 fx using 6 MV photons or a combination with 10 MV photons. Treatments were performed as sliding-window intensity-modulated radiotherapy (IMRT) via photon beams or via volumetric (partial) arc therapy (VMAT). The selection of the irradiation technique was based on the individual patient anatomy, with due consideration of the target volume coverage, dose homogeneity, and sparing of organs at risk. A sequential tumor bed boost of 16 Gy with 2 Gy/fx was performed in those patients up to the age of 50 years, with close or positive resection margins or with high-grade tumors. WBI was performed in a supine position on a Varian True-Beam STx (Palo Alto, CA, USA) linear accelerator. Treatment of left-sided breasts was administered using deep-inspiration breath-hold. For skin-care purposes, patients were instructed to use 5% urea lotion (Eucerin UreaRepair PLUS Lotion 5% Urea, Beiersdorf AG, Hamburg, Germany) two times daily during the WBI treatment period.

### 4.2. Visual Evaluation of Radiation-Induced Dermatitis (RID) and Spectrophotometry (SP)

Radiation-induced dermatitis (RID) assessments were reported corresponding to the CTCAE v4.03 at treatment completion and in the first follow-up visit after two weeks. To prevent a potential grading variability between different observers, only the treating radiation oncologist visually assessed the skin toxicity in his/her patients. Additionally, patients completed a questionnaire on their most severe experience of itching, burning, pain, pigmentation changes, incapacity for work, and limitations in everyday activities related to their acute skin reactions. Therefore, we designed a modified version of the Radiation-Induced Skin Reaction Assessment Scale (RISRAS); patients scored the symptom severity as follows: 0 = not at all, 1 = a little, 2 = quite a bit, 3 = very much.

One day after the last WBI fraction and again after two weeks, ten erythema readings were obtained within the treatment area (two in each breast quadrant and one at the center intersection points of the upper and lower quadrants including the inframammary folds). The CR-10 Plus reflectance spectrophotometer (Konica Minolta, Marunouchi, Japan) was used, which was applied to the skin region of interest avoiding any pressure. The measurement is initiated by illuminating the skin and detecting the reflecting light. Postprocessing using multiple photocells and a build-in microcomputer concludes the procedure and provides a distinct value within the L*a*b color space. The output data is based on the CIE (Commission Internationale de l’Eclairage) system of tristimulus values using the L*a*b* coordinate system. The brightness of the skin is described by the L* parameter ranging from 0 (dark) to 100 (light). a* values enumerate the skin color from green (negative) to red (positive), whereas the b* value provides precise information regarding the blue (negative) to yellow (positive) color axis.

### 4.3. Statistical Analysis

For bivariate correlations, simple linear regressions were calculated for pairs of distinct parameters. The goodness of fit of the linear regression line was determined by R^2^ and the statistical slope deviance from zero was described by the according *p*-value. For comparison between two groups of non-intraindividual distinct numeric parameters, unpaired *t*-tests (univariate) were performed resulting in P values. This approach was chosen, omitting the Welch or Mann–Whitney approach, as the identified data presumably yielded comparable standard deviations and normal distributions. For intraindividual distinct numeric parameters, paired *t*-tests (univariate) were conducted when applicable. If a statistical analysis was preceded by the grouping of data according to discrete or non-discrete parameters, subsequent *t*-tests must be considered bivariate. For breast volumes, a skew value-to-effect pattern (with the effect being RID severity) was identified using FlowJo 10 (Beck and Dickenson) leading to arbitrary categorization for breast volumes into (1) <400 mL (2) 400–800 mL (3) >800 mL. A depiction of the skew value-to-effect distribution pattern is provided in Figure S1. In the case of three or more groups, univariate One-way-ANOVA testing was applied to calculate P values. In the case of one-way-ANOVA, prior data grouping also led to bivariate analyses. Significance levels in all analyses were defined as follows: * for *p* < 0.05, ** for *p* < 0.01, *** for *p* < 0.001 and **** for *p* < 0.0001.

## 5. Conclusions

The portrayed series yields the largest objective data analysis of SP-assessed RID during WBI. SP measurement values were correlated with physician-assessed CTCAE and, for the first time, with a total of 24 RT parameters, patient characteristics, and patient-reported symptoms. We were thus able to scrutinize prior works conducted in this field of research.

Objective and significant risk factors for RID were the breast volume and the use of SW-IMRT instead of VMAT; a sequential boost administration also showed a trend towards a slightly more severe RID. Due to the fact that these results are based on both physician-evaluated and objective RID measurements, they may contribute to the inconsistent or even contradictory debate on possible risk factors for RID.

Although CTCAE gradings strongly correlated with objective SP measurements, future RID research should endeavor to meet the requirement for a more objective approach in the RID evaluation process to augment visual examinations and overcome intra- and inter-evaluator bias in RID assessments. Our set up reference values may, therefore, serve as a solid benchmark and facilitate further work on this important research topic.

## Figures and Tables

**Table 1:** Baseline patient, tumor, and treatment characteristics.

Characteristic	Parameter	Value
Gender		
	Female	142
Age [a]		
	Mean ± SD	59.7 ± 11.0
	Range	19–84
BMI		
	Mean ± SD	26.8 ± 4.5
	Range	18–45.7
	BMI < 20	7
	BMI 20–25	30
	BMI 25–30	80
	BMI 30–35	21
	BMI > 35	4
Breast volume		
[mL]	Mean ± SD	623.6 ± 347.6
	Range	124–1771
	Vol. > 400 mL	30.65%
	Vol. 400–800 mL	42.74%
	Vol. > 800 mL	26.61%
Ethnicity		
	Caucasian	142
Initial L* values		
	Mean ± SD	67.2 ± 4.81
	Range	46.8–76.5
	Initial L* < 65	22.40%
	Initial L* 65–70	52.20%
	Initial L* > 70	25.40%
T Classification (TNM)		
	DCIS	18
	T1	92
	T2	32
Radiation therapy		
	50 Gy in 25 fx	71
	40.05 Gy in 15 fx	71
	Seq. boost of 16	
	Gy in 8 fx	67
	Boost vol. [mL]	
	Mean ± SD	200.4 ± 107
	Range	40–505
	HI_(D2%–D98%)/D50%_	0.144 ± 0.04
	HI_RTOG (max. isodose in_	
	_target/reference isodose)_	1.07 ± 0.03
	rel. D_2%_ [%]	94.29 ± 14.43
	rel. D_50%_ [%]	90.56 ± 13.75
	rel. D_98%_ [%]	81.24 ± 12.88
Treatment technique		
	Sliding-window	115
	VMAT	7

Abbreviations: WBI = Whole-Breast Irradiation; BMI = Body Mass Index; TNM = TNM Classification of Malignant Tumors; D_mean_ = Mean dose within the target volume; HI_(D2%–D98%)/D50%_ = Homogeneity index [(D_2%_–D_98%_)/D_50%_]; HI_RTOG_ = Homogeneity index according to the Radiation Oncology Therapy Group; rel. D_2%_, rel. D_50%_, rel. D_98%_ = Dose received by 2%, 50%, or 98% of the target volume relative to the prescribed dose, respectively; sliding-window = Intensity-modulated Radiotherapy (as sliding-window); VMAT = Volumetric (partial-) arc therapy.

**Table 2:** Distribution of maximum L* and a* values for RID gradings according to CTCAE.

Characteristic	Parameter	L* Max	a* Max
CTCAE 0°	Mean ± SD	67.35 ± 2.44	9.11 ± 1.68
	Median	67.0	9.1
	95% CI	65.9–68.8	8.1–10.1
	Range	64.8–73.6	4.8–11.2
CTCAE I°	Mean ± SD	62.55 ± 4.78	10.95 ± 2.18
	Median	64.0	11.0
	95% CI	61.0–64.1	10.3–11.6
	Range	48.1–70.1	8.18–16.8
CTCAE II°	Mean ± SD	59.47 ± 6.08	12.68 ± 2.01
	Median	59.6	12.84
	95% CI	57.7–61.3	12.1–13.3
	Range	32.9–67.7	10.1–19.4
CTCAE III°	Mean ± SD	53.4 ± 4.29	19.32 ± 1.97
	Median	54.0	18.56
	95% CI	49.6–57.2	17.6–21.1
	Range	46.3–57.7	17.6–22.5

Abbreviations: RID: Radiation-induced dermatitis; CTCAE = Common Terminology Criteria for Adverse Effects; SD = standard deviation; CI = Confidence interval; L* max = maximum L value (spectrophotometry); a* max = maximum a value (spectrophotometry).

**Table 3:** Correlation analyses for multiple parameters in accordance to CTCAE score, maximum L* value and maximum a* value. Values indicate *p*-values for the slope deviances of respective linear regressions, a + or − (in brackets) indicates whether the regression was positive or negative. Values in bold indicate statistically significant differences (*p* < 0.05).

Parameter	CTCAE	L* Max.	a* Max.
Age [a]	0.1289 (−)	0.0825 (+)	0.5491 (**−**)
BMI [kg/m^2^]	0.2282 (+)	0.9428 (−)	0.1212 (+)
Breast volume [mL]	**0.0047 (+)**	0.9379 (+)	0.0509 (+)
Boost (y/*n*)	0.0635 (+)	0.5084 (−)	0.9629 (+)
Boost vol. [mL]	0.6119 (+)	0.6456 (−)	0.4453 (+)
Mean Dose to PTV [%]	0.4435 (−)	0.9059 (−)	0.9889 (+)
rel. D_2%_	0.5635 (−)	0.1062 (−)	0.1147 (+)
rel. D_50%_	0.5532 (−)	0.1087 (−)	0.1210 (+)
rel. D_98%_	0.5595 (−)	0.1011 (−)	0.1178 (+)
V_≥107%_ [mL]	0.8424 (+)	0.5715 (−)	0.9127 (+)
HI_RTOG_	0.8209 (+)	0.8924 (+)	0.3468 (+)
HI_(D2%–D98%)/D50%_	0.7125 (+)	0.7217 (+)	0.7711 (+)
L* baseline	0.2848 (−)	**<0.0001 (+)**	**0.0155 (−)**
a* baseline	0.2892 (+)	**<0.0001 (−)**	**0.0006 (+)**
b* baseline	0.3702 (−)	**0.0002 (−)**	0.5846 (+)
CTCAE		**<0.0001 (−)**	**<0.0001 (+)**
L* max.	**<0.0001 (−)**		**<0.0001 (−)**
a* max.	**<0.0001 (+)**	**<0.0001 (−)**	
b* max.	0.5229 (−)	0.1258 (−)	0.4628 (+)
Pain (RISRAS)	**<0.0001 (+)**	**0.0115 (−)**	**0.002 (+)**
Burning (RISRAS)	**0.0003 (+)**	**0.025 (−)**	0.0856 (+)
Itching (RISRAS)	0.1517 (+)	0.8369 (−)	0.7335 (−)
Activities (RISRAS)	**<0.0001 (+)**	**0.0011 (−)**	**0.0409 (+)**

Abbreviations: BMI = Body Mass Index; IMRT = Intensity-modulated Radiotherapy (sliding-window); VMAT = Volumetric (partial-) arc therapy; PTV = Planning Target Volume; V_≥107%_ = Volume receiving ≥ 107% of prescribed dose; HI_RTOG_ = Homogeneity index (Radiation Oncology Therapy Group); HI_(D2%–D98%)/D50%_ = Homogeneity index [(D2%–D98%)/D50%]; CTCAE = Common Terminology Criteria for Adverse Effects; RISRAS = Radiation Induced Skin Reaction Assessment Scale; rel. D_2%_, rel. D_50%_, rel. D_98%_ = Dose received by 2%, 50%, or 98% of the target volume relative to the prescribed dose, respectively.

## Supplementary Materials

The following are available online at https://www.mdpi.com/2072-6694/12/9/2444/s1, Figure S1: Skew value-to-effect distribution pattern for breast volume and maximum a* values.

## Abbreviations

RID	Radiation-Induced Dermatitis
RT	Radiotherapy
Gy	Gray (Unit)
SP	Spectrophotometry
CTCAE	Common Terminology Criteria for Adverse Effects
RTOG	Radiation Therapy Oncology Group
EORTG	European Organization for Research and Treatment of Cancer
L* max	maximum L value (spectrophotometry)
a* max	maximum a value (spectrophotometry)
b* max	maximum b value (spectrophotometry)
RISRAS	Radiation-Induced Skin Reaction Assessment Scale
fx	Fraction(s)
CF	Conventional Fractionation
HF	Hypofractionation
SD	Standard Deviation
WBI	Whole Breast Irradiation
BMI	Body Mass Index
TNM	TNM Classification of Malignant Tumors
DCIS	Ductal Carcinoma In Situ
HI_(D2%–D98%)/D50%_	Homogeneity Index_(D2%–D98%)/D50%_
HI-RTOG	Homogeneity Index (Radiation Therapy Oncology Group)
SW	Sliding Window
VMAT	Volumetric (partial-) arc therapy
IMRT	Intensity-modulated Radiotherapy (sliding-window)
D_mean_	Mean dose within the target volume
rel. D_2%_	Dose received by 2% of the target volume relative to the prescribed dose
rel. D_50%_	Dose received by 50% of the target volume relative to the prescribed dose
rel. D_98%_	Dose received by 98% of the target volume relative to the prescribed dose
V_≥107%_	Volume receiving ≥ 107% of prescribed dose
ANOVA	Analysis Of Variance
