# Supplementary material for: Objective Evaluation of Risk Factors for Radiation Dermatitis in Whole-Breast Irradiation Using the Spectrophotometric L*a*b Color-Space
Source: Cancers (Basel). 2020 Aug 28;12(9):2444. doi: 10.3390/cancers12092444 (PMC7563751; doi:10.3390/cancers12092444)
Supplement: Supplementary file 1 [file cancers-12-02444-s001.pdf]

Supplementary Materials: Objective Evaluation of Risk Factors for Radiation Dermatitis in Whole-Breast Irradiation Using the Spectrophotometric L*a*b Color-Space

Alexander M. C. Böhner, David Koch, Frederic Carsten Schmeel, Fred Röhner, Felix Schoroth, Gustavo R. Sarria, Alina-Valik Abramian, Brigitta Gertrud Baumert, Frank Anton Giordano and Leonard Christopher Schmeel

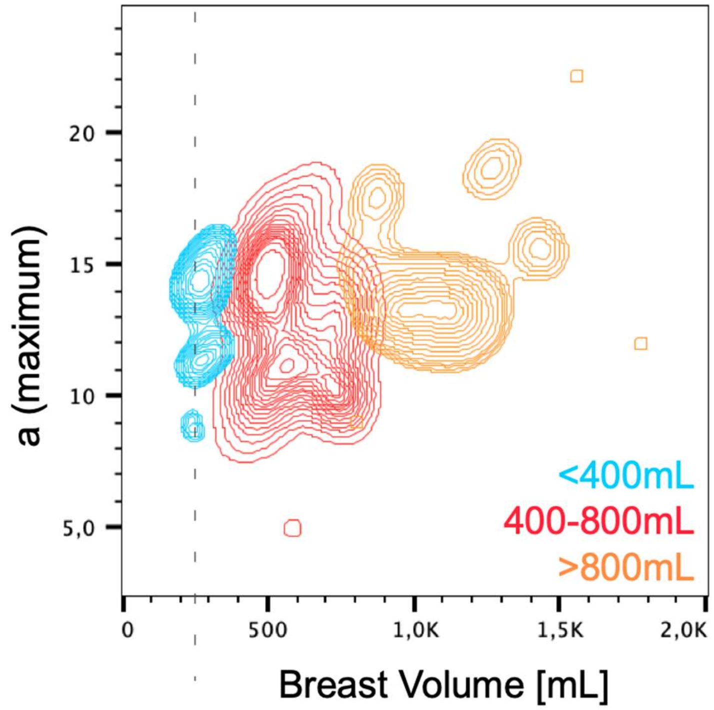

Figure S1. Skew value-to-effect distribution pattern for breast volume and maximum a* values. Subgroups are colored in blue (breast volume < 400 mL), red (breast volume 400–800 mL) and orange (breast volume > 800 mL). Contour blot with isodose-lining at 5%.

© 2020 by the authors. Licensee MDPI, Basel, Switzerland. This article is an open access article distributed under the terms and conditions of the Creative Commons Attribution (CC BY) license (http://creativecommons.org/licenses/by/4.0/).
